# A novel wheat lodging resistance evaluation method and device based on the thrust force of the stalks

**DOI:** 10.1371/journal.pone.0224732

**Published:** 2019-11-12

**Authors:** Suwei Feng, Dechuan Kong, Weihua Ding, Zhengang Ru, Gan Li, Liyuan Niu

**Affiliations:** 1 Wheat Center, Henan Institute of Science and Technology, Xinxiang, China; 2 College of Life Science and Technology, Henan Institute of Science and Technology, Xinxiang, China; 3 College of Artificial Intelligence, Henan Institute of Science and Technology, Xinxiang, China; Institute of Genetics and Developmental Biology Chinese Academy of Sciences, CHINA

## Abstract

Wind speed is the most essential factor causing wheat lodging. Accurate understanding of the wind speed characteristics at near-surface layer of wheat fields and its effect on lodging is the basis of objective evaluation of wheat lodging resistance. In this paper, the characteristics of wind speed at the near-surface layer of wheat fields and their impact on lodging were studied. A new device was proposed for directly measuring the critical thrust force of wheat population lodging resistance in the field based on the black box method. A novel wheat stem lodging resistance evaluation method/model was established based on the critical thrust force of wheat population stem lodging and the wind speed characteristics of field near-surface layer. The method used the lodging critical wind speed as the index of wheat lodging resistance, which was verified by wind tunnel and field experiment. The results showed that there was a significant positive correlation between the critical wind speed of wheat lodging resistance and its critical thrust force. The values of wheat canopy apparent roughness length, wind attack angle, ventilation coefficient and other wind field characteristics had important effects on the calculation of wheat lodging resistance critical wind speed. The method can eliminate bias when calculating wheat lodging resistance by considering only one or a few indicators and the results of field lodging evaluation were consistent with those of field lodging survey. The method is simple and can be used to assess the lodging resistance of wheat, select extension regions for wheat varieties, and evaluate lodging factors in the field.

## Introduction

Lodging is a state of permanent dislocation of cereal stems from their upright position[1,2]. Lodging is divided into root lodging and stem lodging, where it can result in significantly reduced grain yields, and cause a decline in grain quality due to bacteria growth and toxin accumulation [3–6]. Many approaches can be used to prevent lodging in wheat, but the simplest and most effective method is to select varieties that are naturally lodging resistant. For a long time, breeding researchers have proposed many methods to evaluate the lodging resistance of wheat, such as the stalk bearing method [7], the stalk breaking method [8], the stalk strength method [9], the artificially inducing lodging of wheat (by dragging a weighted apparatus across plots at the early milk stage) [10], as well as the mechanical analysis method of wheat stalk [11]. These methods have played an important role in the selection of lodging resistant varieties. However, as wheat lodging resistance depends on many factors, and there are also complex constraining relationships between these factors, such as plant height, internodes length, thickness of stem, the stem’s chemical composition and mechanical strength, planting density, growth period, etc. [3,12–19]. Therefore, it is still a serious challenge to provide an integrated estimate of the actual lodging resistance of wheat varieties. Moreover, and most importantly, many of these methods have not considered the effect of wind speed on lodging, which is a key natural factor that causes lodging. In recent years, the research of wheat lodging has mainly focused wheat-lodging mechanisms, wheat-lodging models, and new wheat-lodging evaluation methods [1–2,20–24]. Baker et al considered the dynamics of the wheat stems and stem structure as a simple damped harmonic oscillator and a columnar structure, respectively, and proposed a model of wheat lodging based on wheat single stem physical characteristics and field wind speed characteristics[1]. The stem lodging occurs because wind causes a bending moment on the stem base that is greater than its stem breaking moment. The model was verified through field trials by Berry et al [2], and its parameters were determined via wind tunnel testing [23].

To reduce the possible deviation of evaluating wheat lodging resistance only by individual or a few factors, and simplify the evaluation procedure, Niu et al. designed a portable electronic instrumment for the critical thrust force (CTF) of crop population in fields with a multi-function tripod, ball guide, digital dynamometer and a specially designed probe. The measuring principle of the instrument is to apply a certain thrust to the center of gravity of the crop stalk in the horizontal direction by using the specially designed probe, and the lodging CTF required to bend the stalk of the plant to 45° is determined by the digital dynamometer. The greater the lodging CTF, the stronger the lodging resistance of the crop. On the basis of this, based on the Bernoulli wind speed and wind pressure conversion principle, a model for calculating the critical wind speed (CWS) was established according to the parameters such as the lodging CTF, the height of the center of gravity, the length of the apparent roughness, and so on[24]. Since the lodging determination method and calculation model used the field wheat population as the evaluation object, it can eliminate bias when calculating wheat lodging resistance by considering only one or a few indicators, as well as the possible complex interactions among these parameters. Therefore, this method and model have been recognized by some breeders since it was proposed [25–31]. However, due to the constraints of meteorological factors, the model is subject to further optimization and verification.

We assume that the lodging is initiated by the continuous oscillation of small wheat population. The downward propagation of wind is the source of external power for wheat lodging. By using the black box method, many factors, which are related to the lodging resistance of wheat but their real effects and their correlation with each other are difficult to be simply analyzed, can be included in a black box. Based on the lodging CTF of the field wheat population as the starting point to evaluate its lodging resistance, not only the results are more accurate, but also the method is simpler. The purpose of this paper is to: (1) study the characteristics of wind speed at the near surface layer of wheat fields, and determine its effect on wheat stem lodging; (2) develop a new fast testing instrument or device for lodging resistance of crops; (3) optimize the model of the stem lodging CWS; and verify the model using wind tunnel simulations and field tests.

## Materials and methods

### Characteristics of wind speed at the ground layer of wheat fields

Wind speeds were continuously measured from April to June in 2014–2016. This three-month period is a key phase where winter wheat is prone to lodging (from flowering to maturity) in northern China. The fields are located in Xinxiang County, Henan Province (N 35o 10' 15.45" E113o 53' 54.11'). Two ultrasonic 3D anemometers (Wind Master, Gill Instruments Ltd., UK) and a PC-2F multichannel anemometer (Jinzhou Yangguang Science and Technology Co., Ltd, China) were used. The ultrasonic 3D anemometers can simultaneously measure wind speed components in X(*μ*_*x*_), Y (*μ*_*y*_), Z (*μ*_*z*_) directions, and the virtual temperature at a sampling frequency of 1–20 Hz. The measurement range of wind speeds is from 0 to 45 m/s at a resolution of 0.01 m/s, and the wind direction can be measured from 0 to 359 degrees at a resolution of 0.1 degrees. Each ultrasonic anemometer was connected to a TYY model data transparent transmission recorder, where the frequency of data recording was 10 Hz, and one file was created for each hour. The 10 m and 2 m from the ground are the heights commonly used for standard wind speed observations in conventional meteorological observations. The wind speed at the height of 2 m and 1 m from the ground is the wind speed most affected by the wheat canopy. In order to study the variation law of 3D wind speed with height, the way of observing wind speed at two heights was adopted. Due to the limitation of the number of ultrasonic anemometers, the heights of the probes used for observation of wind speed characteristics in different years are different. The installation data of 3D anemometers in different years are shown in Table 1.

**Table 1:** Installation data of the ultrasonic 3D anemometers.

ID	Probe height (m) in 2014	Probe height (m) in 2015	Probe height (m) in 2016
1	2.00	1.09	2.00
2	10.00	2.08	6.00

Note: The probe height is the height of the center of the ultrasonic 3D anemometers probe from the ground.

An EC-9S digital wind speed sensor was used in the multi-channel observation system, which measured the full-scale wind direction at a resolution of 1 degree, and speeds ranging from 0 to 75 m/s at a resolution of 0.1 m/s. The data were automatically recorded by each channel every minute, and the device could trace the instantaneous value as well as the average over the previous two minutes. The installation height of the multi-channel anemometer probe No. 1–6 was 1.1m, 2.24m, 4.2m, 6.2m, 8.2m and 10.2m respectively. The continuous observation times were from May 19 to June 12, 2015.

**Fig 1:**
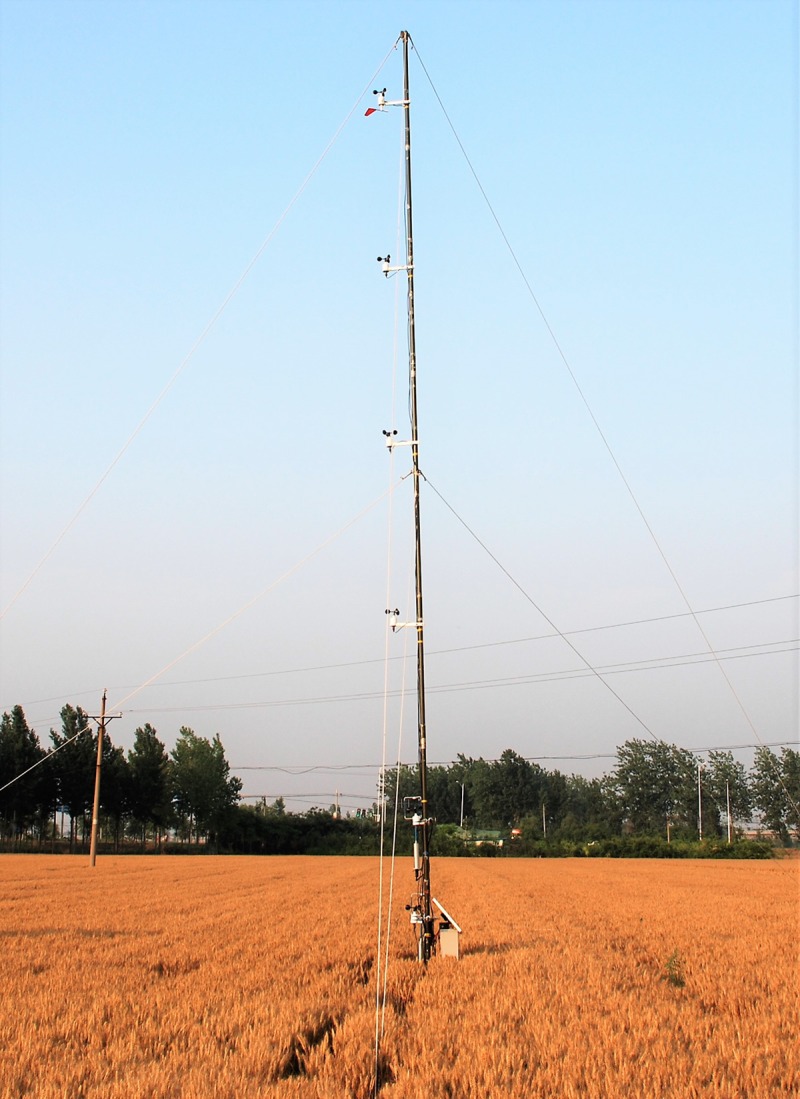
Wind speed characteristics at the near surface layer of the wheat field (May 2015).

The ultrasonic 3D anemometers and multi-channel observation system were powered by solar energy (Fig 1). A total of 340 hours of the wind speed and direction at the six heights were obtained from May 19 to June 12, 2015.

### Processing the wind speed data

The *μ*_*x*_, *μ*_*y*_, *μ*_*z*_, wind directions and virtual temperatures collected by the 3D anemometers were grouped in intervals of 10 minutes, and processed to remove outliers. The outliers were removed via the absolute value average method[32]. The sequence data (*x*_*i*_) of dynamic signals exhibited variation within a certain threshold (*w*). If |*x*_*i*_|≥*w*, *x*_*i*_ was considered as an outlier of *x*(*t*) and was removed. The deleted data points were replaced by the absolute value of the average:

$$w=k(\frac{1}{n}\sum \left|x_{i}\right|)=k\left|{\bar{x}}_{i}\right|$$ (1)

$$\left|x_{i}\right|=\frac{1}{n}\sum \left|x_{i}\right|$$ (2)

Where *k* is an empirical value coefficient, generally 4–5, where a value of 4 is used in this paper.

The wind speeds and directions recorded by the multi-channel observation system were used directly without pre-processing.

### Statistical analysis

The lodging CTF of wheat population reported in this paper were the means of 10 individual measurements. The mean value, standard deviation, frequency distribution, correlation analysis and significance analysis of experimental data listed in this paper were all carried out by using SPSS 13.0 statistical software (SPSS Inc., Chicago, IL).

#### Atmospheric stability at the ground layer of wheat fields

The 10-minute average of *μ*_*x*_, *μ*_*y*_, and *μ*_*z*_ and that of the horizontal wind speed, *U*, were calculated using Xu et al methods[33]. The atmospheric stability was obtained by examining the air temperature [34–35] and wind speed recorded by the 3D anemometers. In this study, the gradient Richardson number (*Ri*) was employed to determine the atmospheric stability at the ground layer [36]:

$$Ri=\frac{g\times \Delta T\times \left(z_{2}-z_{1}\right)}{\bar{T}\times {\left(\bar{\mu_{2}}-\bar{\mu_{1}}\right)}^{2}}$$ (3)

Where $\bar{T}$is the mean temperature of air (K), which was calculated using: $\bar{T}$ = 273.16 + (*T*_*2*_*—T*_*1*_)/2. Here $\Delta \bar{T}$represents the difference in air temperature between two observation heights, *z*_*1*_ and *z*_*2*_ indicate the installation heights of two anemometers, and *g* is the acceleration due to gravity, 9.8 m/s^2^. *Ri* > 0, *Ri* = 0, and *Ri* < 0 indicate that the atmospheric stratification is in a stable state, a neutral state, and an unstable state, respectively.

#### Wind speed profile and the apparent roughness length

Since the lodging occurs on cloudy or rainy weather, it is necessary to study the effects of weather conditions on the daily variation of wind speed profile and apparent roughness length. The dataset obtained with the ultrasonic anemometers and the multi-channel anemometers were analyzed for two weather conditions—sunny and cloudy—for 24 hours. The variation in the wind speed profile and the apparent roughness with time and weather conditions was analyzed. (1) Wind speed data at six heights, as obtained by the multi-channel anemometers, were ranked according to that measured by the wind speed probe at 10 m. (2) The data were segmented based on a step size of 0.5 m/s, where the average wind speed at each section was calculated. (3) Wind speed profile was plotted by using 6 observation heights and corresponding wind speed, and the apparent roughness of the wheat field was calculated by extrapolating the wind profile curve to the coordinate axis (i.e. for an average wind speed of zero). (4) The curve of roughness versus wind speed was drawn by using the wind speed at a height of 10 m from the whole day and corresponding apparent roughness, and the best-fitting equation was determined by the least squares method.

#### Wind attack angle at the ground layer of wheat fields

Since the wind direction is rarely parallel to the ground, the angle of attack, *θ*, describes the difference between the wind direction and the surface. When wind blows upward, the wind attack angle is positive, and vice versa [37]. The attack angle was determined as Xu et al[33]):

$$\theta =\arctan\frac{{\bar{\mu }}_{z}}{U}$$ (4)

To study the variation of wind attack angle with weather condition and observation time, the 3D wind speed data of sunny, cloudy and cloudy with light rain weather were used to calculate the wind angle of attack. The calculation steps of the wind attack angle as follows. (1) Wind speed data from the whole day with a maximum daily average wind speed greater than 8.0 m/s were segmented by 10 minutes. (2) Three-second moving averages of the wind attack angle in each 10-minute period were calculated. (3) The maximum and minimum wind angles of all samples were sorted, and the mean of the top 25% of the sequence was taken as the wind attack angle of the model.

### Determination of the lodging CTF of wheat population

**Fig 2:**
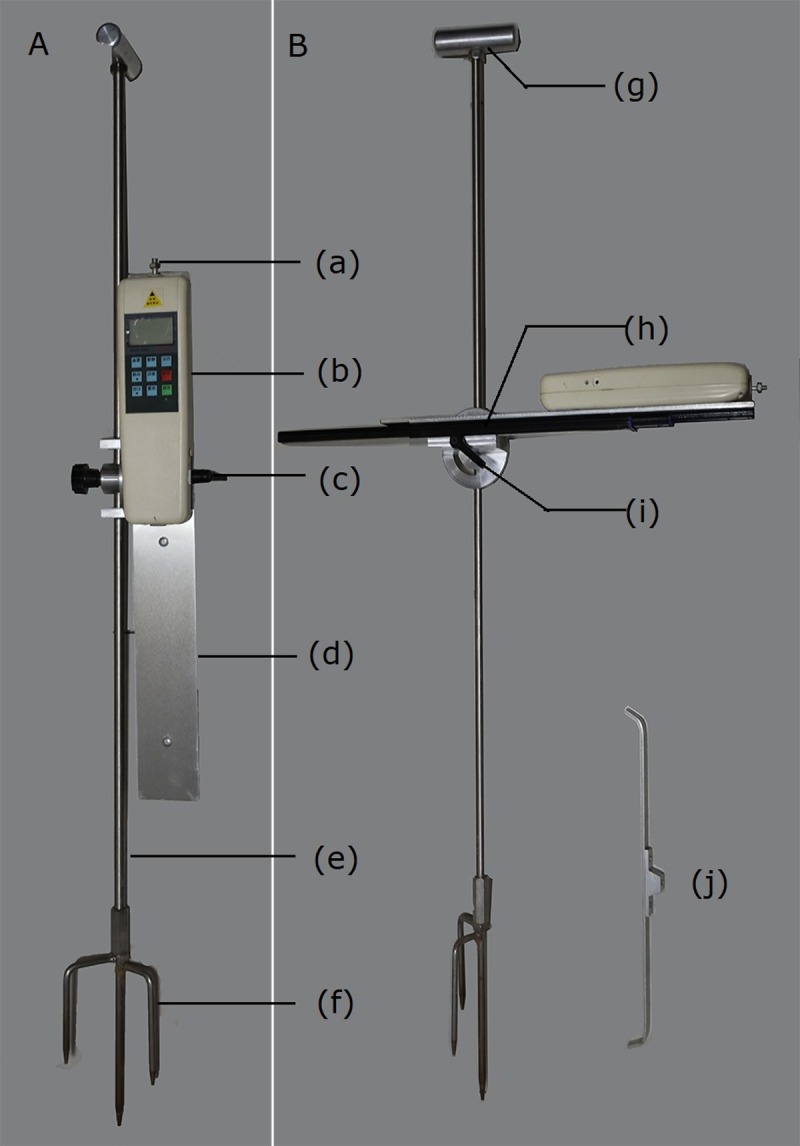
The rapid measuring instrument for crop lodging resistance. A. Instrument folding state. B. Instrument unfolding state (working state). Composition and name: (a) probe mounting position; (b) dynamometer; (c) connecting plate locking handle; (d) dust-proof connecting plate; (e) circular ruler rod; (f) three tooth steel fork; (g) circular ruler rod handle; (h) ball guide way; (i) rotary positioning plate; (J) population lodging thrust force probe.

The lodging CTF of wheat population was measured by a specially designed "Rapid measuring instrument for crop lodging resistance"(Patent No.ZL2014 1 0417667.1). The instrument was mainly composed of dynamometer, dust-proof panel, ball guide way, connecting plate, rotary positioning plate, circular ruler rod, three tooth steel fork and probes. The dynamometer was designed by using the existing digital push-pull meter circuit, and was used to measure the CTF of crop lodging. The basic measurement principle was to exert a force on the wheat population stalk horizontally by the probe of the dynamometer, and measure the thrust force when the population stalk was bent to a 45° from the perpendicular. The greater the lodging CTF, the stronger the crop stalk lodging resistance. The ball guide was used for the dynamometer moving smoothly and slowly in the horizontal direction. Canopy height scale and height scale of the center of gravity on the circular rod were used to measure the height of the crop canopy and the height of the crop center of gravity, respectively. The rotary positioning disc was used for the conversion of the horizontal and vertical positions of the dynamometer. The three tooth steel fork was used for rapid positioning and fixing of instruments in dry land and paddy fields. The instrument can be used to determine the stem breaking strength of wheat, rice, millet, and these crops’ lodging critical thrust force. The structure and composition of the instrument are shown in Fig 2.

The test procedure for the lodging CTF was as follows. (1) The special U-shaped probe for measuring the lodging CTF of population wheat was installed to the front of the dynamometer. (2) The instrument was vertically fixed to the position of about 0.25 m in front of the tested wheat population by three tooth steel fork. (3) The ball guide was rotated clockwise 90°, and the connecting plate locking handle was tightened to keep the dynamometer horizontal. (4) Crop canopy height was measured by using the height scale on the circular ruler rod, and the dynamometer was adjusted to the height of the center of gravity of the canopy (about 2/3 of the canopy height) by the height scale of the crop center of gravity. (5) The peak measurement mode was selected and the ‘zero’ button pressed to make the value displayed on the measurement unit read ‘zero’. (6) The dynamometer was pushed horizontally along the ball guide way to measure the thrust force when the population stalk was bent to a 45° from the perpendicular (N m^–1^). Each sample was measured 10 times and a mean value calculated.

### Lodging CWS model and experimental verification

#### Lodging CWS model for wheat population

Basic principle of the model for calculating the lodging CWS of wheat population is that the lodging of wheat is caused by the load exerted vertically on the stalk of wheat population is greater than the maximum load that the stem base can bear. Field wheat population is taken as the observation object, the lodging CTF is firstly measured; then the ground equivalent lodging (EWS) is calculated according to the Bernoulli wind speed and wind pressure conversion principle and the wheat canopy parameters; and finally, it is converted into the standard wind speed at 10 m above the ground according to the near-surface layer wind speed profile characteristics of wheat field. The basic calculation procedure is as follows.

(1) Determine the lodging CTF of a wheat population, that is, the thrust force of the plant was pushed to a 45° from the ground (N/m^-1^).
(2) Calculate the critical wind load of the wheat population *W*_0_ (1000 N/m^2^):

$$w_{0}=\frac{k_{p}\times P}{L\times (h_{c}-z_{0})\times 1000}$$ (5)

Where *P* is the lodging CTF. *L* is the probe length (0.33 m). *K*_*P*_ is the resistance coefficient of the probe, which is 0.75–1.0 depending on the resistance of the probe, and 0.75 in this paper. *h*_*c*_ is the height of the wheat canopy. *z*_0_ is the apparent roughness length of the wheat field.

(3) Calculate the corresponding lodging EWS (ν_0_):

$$w_{0}=\frac{r\times v_{0}{}^{2}}{2g}=\frac{0.01225\times v_{0}{}^{2}}{2\times 9.8}=\frac{v_{0}{}^{2}}{1600}$$ (6)

Substitute formula (5) into formula (6) and get formula (7):

$$v_{0}=\sqrt{\frac{1600\times k_{p}\times P}{L\times (h_{c}-z_{0})\times 1000}}$$ (7)

(4) Calculate the ground equivalent lodging EWS (ν_0_^’^) (m/s), that is, the wind speed at a specific height that can penetrate the canopy and cause lodging.

$$v_{0}^{\prime }=\frac{1}{\left(1-\alpha \right)}\times \cos\theta \times \beta \times k\times \sqrt{\frac{1600\times k_{p}\times P}{L\times (h_{c}-z_{0})\times 1000}}$$ (8)

Where α is the ventilation coefficient. It is equal to the wind speed at the front of the population minus the wind speed at the back of the population for the same height, and it depends on the number of main stems per unit area in the wheat population. *β* is the gust factor. And *k* is the adjustment coefficient of the lodging CWS, generally 0.85.

(5) Calculate the lodging CWS ν_10_ (m/s), this is, the standard wind speed at 10 m above ground (Instantaneous extreme wind speed):

$$v_{10}=v_{0}^{\prime }\times \left(\frac{\ln10-\ln z_{0}}{\ln(1+h_{c})-\ln z_{0}}\right)$$ (9)

#### Wind tunnel simulation verification of the lodging CWS model

A wind tunnel simulation of wheat lodging resistance was carried out at the laboratory of the Henan Institute of Science and Technology in Xinxiang City, Henan from 2013 to 2015. Eleven wheat varieties (*Triticum aestivum* L.) with different lodging resistance were used as experimental materials: Yumai 18, Zhoumai 18, Aikang 58, Pingan 6, Zhengmai 9023, Bainong 418, Bainong 419, Zhonglian 2, Zhoumai 26, and Caizhi 9333. All of them were provided by the Wheat Center of Henan Institute of Science and Technology. The experimental material was divided into two periods in 2013–2014; the first planting was sown on October 10, 2013, and the second was sown on October 20, 2013. The experimental material was sown on October 10, 2014 for the 2014–2015 periods. The plants were grown in large plastic planting boxes of dimensions 0.60 m×0.40 m×0.25 m, following normal management practices.

The experiment section of the wind tunnel was 7.00 m×1.20 m×2.00 m, where a transducer was used to adjust the wind speed. The wind speed profile of the wind tunnel was similar to that of the ground layer in the field, and the wind speed increased with elevation following a logarithmic function. A horizontal clapboard was installed at 0.5 m above the bottom of the wind tunnel with a tilt board in front of it, which allowed wind to blow along the clapboard to the wheat population stalks 0.16 me above the ground. This was used to simulate the effect of strong wind on the lodging at an apparent roughness of 0.16 m.

**Fig 3:**
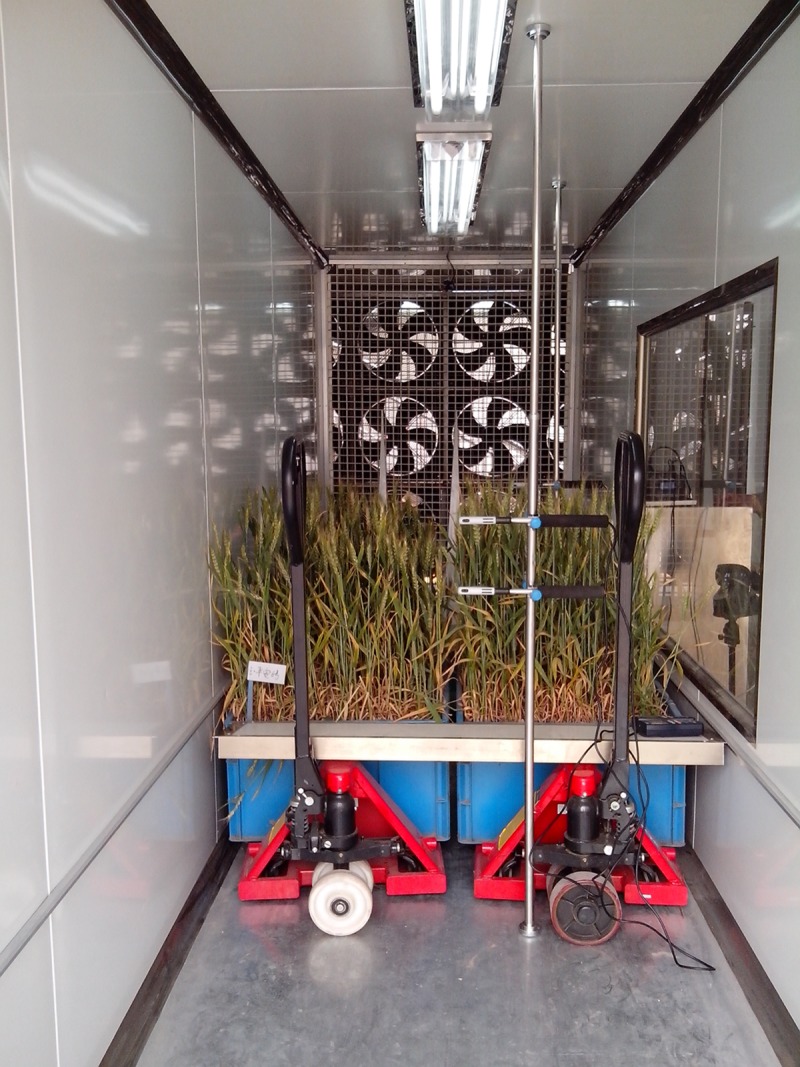
Wind tunnel simulation of lodging resistance in wheat.

Determination of the lodging CWS was carried out when wheat was at the mid-grain filling stage. Wheat materials used in the wind tunnel simulation experiments were first used to determine the lodging CTF by the rapid measuring instrument for crop lodging resistance, and the height of the wheat canopy was recorded at the same time. The wheat growing boxes were then moved into the wind tunnel, two at a time. They were placed side-by-side vertical to the direction of the wind. Two hot wire anemometer probes were installed horizontally in front of, and behind, the wheat population at 2/3 of the height of the canopy (another two anemometers were installed above the canopy). The wind speed was gradually increased until the plants tilted back at a 45° angle, whereafter the wind speed in front of and behind the population was recorded (Fig 3). The experiments were repeated three times, and their average was taken as the lodging EWS. This was then compared with the calculated wind speed based on the lodging CTF.

#### Field experiment verification of the lodging CWS model

Six wheat varieties were used in the field trial: Zhoumai 18, Aikang 58, Zhoumai 26, Bainong 418, Bainong 419, and Zhonglian 2. All of them were provided by the Wheat Center of Henan Institute of Science and Technology. The field experiments were performed at the wheat test field of Henan Science and Technology College, Xinxiang County, Henan Province from October 2013 to June 2014 and from October 2014 to June 2015. A randomized block design was implemented with three replicates. The plots were 4 m long with 10 lines, where the space between the lines was 0.23 m, which were managed according to general practice in the field.

The experiments began at the flowering stage until complete maturation, and data were measured every seven days. The lodging CTF was determined 10 times for each population. The critical wind load, the lodging EWS, and the lodging CWS were sequentially calculated.

## Results

### Basic characteristics of the wind field at the ground layer

#### Atmospheric stability on wheat fields

**Fig 4:**
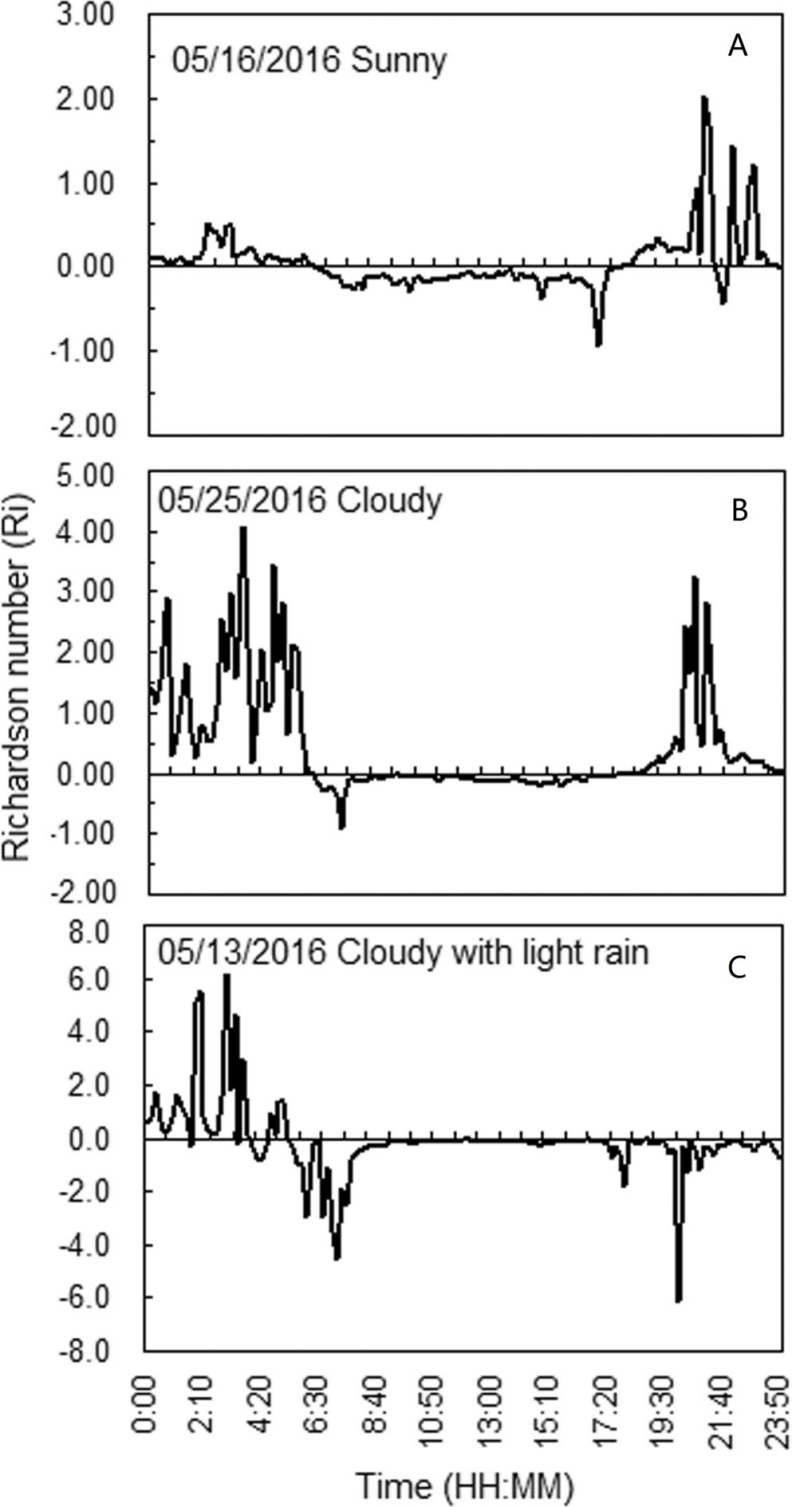
Variation in the Richardson number and the atmospheric stability at the near-surface layer of wheat field.

The stability of the atmosphere is related to the observation time and weather conditions. javascript:void(0);Cloudy days and rainfall are the main weather causing wheat lodging. In order to fully understand the variation of atmospheric stratification stability with time under different weather conditions, the variation of atmospheric stratification stability with time under sunny, cloudy and rainy weather conditions was studied. The pattern of variation in the Richardson number versus time and the three weather conditions—sunny, cloudy, and cloudy with rainy—is shown in Fig 4.

Under sunny and cloudy conditions, the atmosphere in the field was mainly stable from 00:00 to 6:30, but exhibited an unstable state from 06:30 to 18:00, and reverted to a stable state from 18:00 to 24:00. The atmosphere maintained an unstable state during strong or persistent winds and rainy days. At the ground layer, a neutral atmosphere only appeared for a short period, around 06:30 or 18:00, which is the transition state from a stable to an unstable state, or vice versa.

#### Wind speed profile and apparent roughness

Under varying weather conditions and wind speeds, the patterns of variation in wind speed at various heights were comparable in the wheat field. Wind speed increased with elevation, where the change followed an exponential or logarithmic distribution. The smallest *R*^*2*^ of the fitted equations was 0.764 and the maximum was 1.000, with an average of 0.969. The profile distribution of the wind speed at the ground layer of the wheat field was independent of atmospheric conditions.

**Fig 5:**
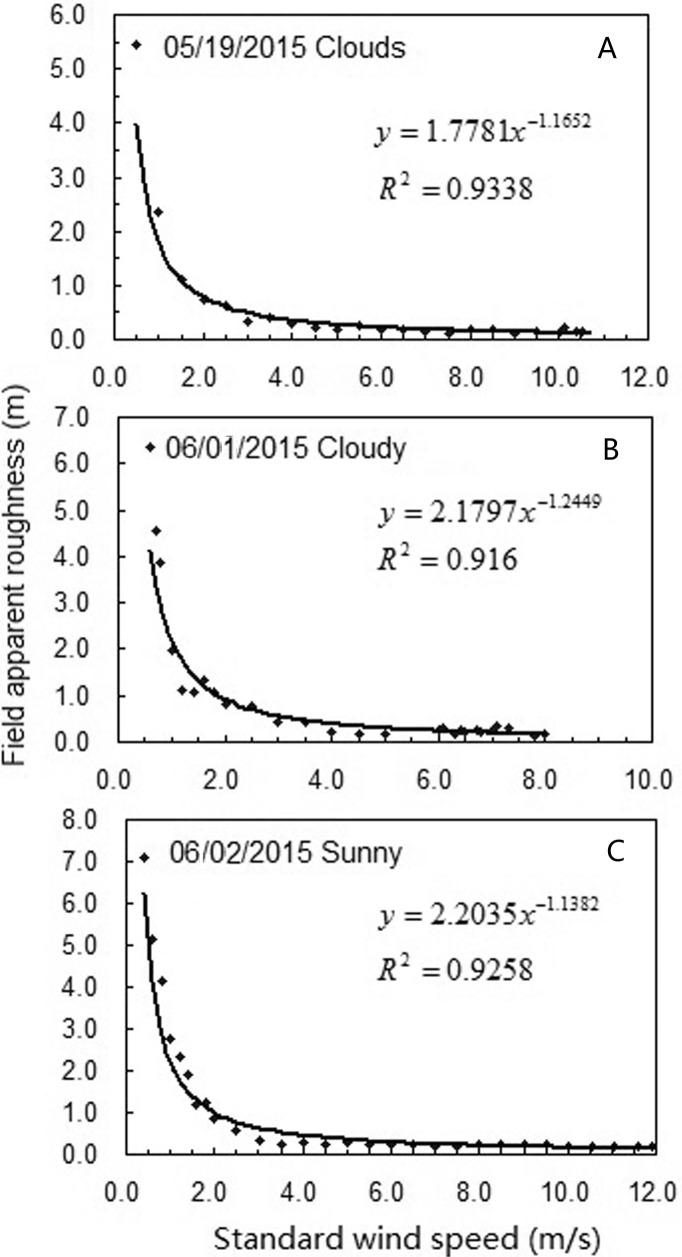
The relationship between the wind speed and the apparent roughness at the near surface layer of the wheat field.

The apparent roughness is related to the weather conditions and wind speed. The relationship between the apparent roughness and the wind speed at the near surface layer of the wheat field is shown in Fig 5.

Under different weather conditions, the apparent roughness of the field linearly decreased with an increase in wind speed, and their relationships were best fit with an exponential function, with correlation coefficients, *R*^2^, ranging from 0.916 to 0.926. In addition, the apparent roughness was also related to the weather conditions. For an 8–10 m/s wind speed, the apparent roughness ranged from 0.10 to 0.16 m in May 19^th^ and June 1^st^ (cloudy), but the apparent roughness ranged from 0.13 to 0.21 m in June 2nd (sunny). The apparent roughness during sunny days was significantly greater than that during cloudy days.

#### Variation in the wind attack angle

**Fig 6:**
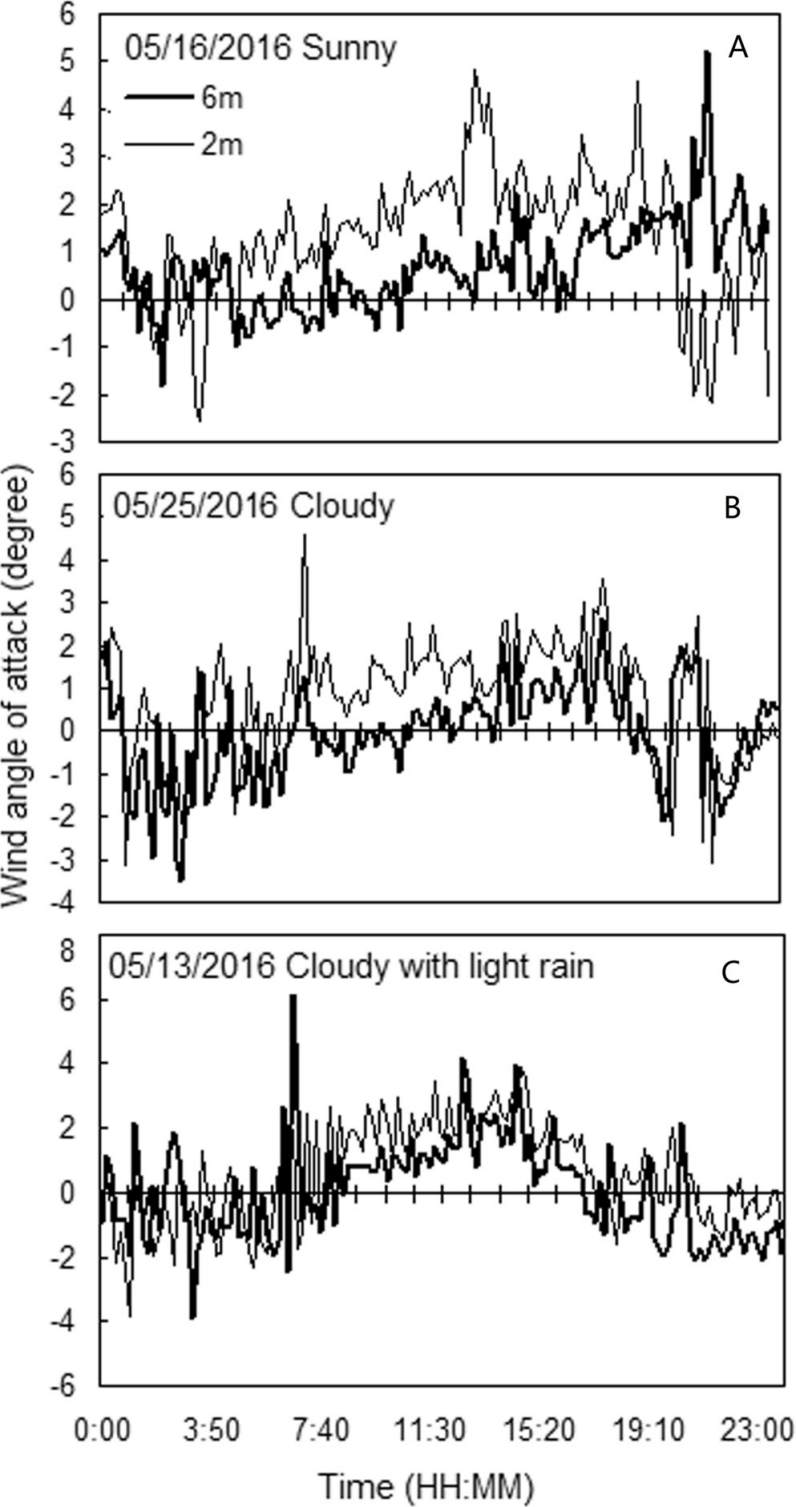
The variation in the 10-minute average of the wind attack angle at the ground layer of the field for various times and weather conditions.

The variation in the 10-minute average of the wind attack angle at the ground layer of the field for various times and weather conditions is shown in Fig 6.

The wind attack angle showed a significant diurnal variation, exhibiting a saddle-shaped curve. In general, the negative attack angle gradually decreased during 0:00–6:30 followed by a positive attack angle from 06:30–18:00, whereafter the negative attack angle gradually increased again. The attack angle and its evolution were related to the observation time, weather, and height. During the evening, the attack angle was generally negative, and it was positive during the day. Compared with that measured during sunny weather, the positive attack angle decreased significantly during cloudy and rainy weather, and the negative attack angle increased significantly.

The variation of wind attack angle showed significant instantaneous characteristics, and its magnitude was closely related to the observation distance. The variation in the three-second average of the maximum wind attack angle under different weather conditions is shown in Table 2.

**Table 2:** Variation in the wind attack angle at the near-surface layer of the field under different weather conditions.

Date of observation and weather conditions	Positive wind attack angle of 3 s		Negative wind attack angle of 3 s	
	Ave	25% Ave	Ave	25% Ave
1 m				
2015-5-21 (Light Rain)	26.27±20.11a	56.01±8.77c	-16.4±11.07a	-31.71±6.60a
2015-5-22(Cloudy)	35.38±14.42b	53.29±5.96c	-14.71±9.69a	-27.37±10.20a
2015-5-23 (Sunny)	23.17±15.03a	44.60±6.90b	-17.52±11.80a	-33.97±9.99a
Average	28.27±16.52B	51.30±7.21B	-16.21±10.85B	-31.02±8.93B
2 m				
2015-5-21(Light Rain)	16.87±13.15a	37.05±10.07b	-11.38±8.41a	-22.17±8.22b
2015-5-22(Cloudy)	18.41±9.70a	30.70±11.76b	-7.56±6.85a	-16.77±7.97ab
2015-5-23(Cloudy)	26.55±15.71b	48.77±5.48c	-16.17±10.11b	-30.21±7.80c
Average	20.61±12.85A	38.84±9.10A	-11.70±8.46A	-23.05±8.00A

Note: The Ave values are the daily average of the 3-second moving average wind angle of attack calculated by 10-minute segments. The postive wind angle of attack is the maximum of the 3-second moving average wind angle, while the negative wind angle of attack is its minimum. "TOP 25% Ave" in the postive wind angle of attack column is the average of the first 25% values in the positive wind angle of attack ZA sequence, while the "TOP 25% Ave" is the opposite in the negative wind angle of attack.

The attack angle decreased with increasing altitude, and the amplitude of the variation at the ground layer was substantially larger than that at higher altitudes. There was a significant difference between the positive and negative attack angles between the two observational heights (*P*<0.01). Although the wind attack angles for the different weather conditions had obvious differences, it was not statistically significant (*P*>0.05). This preliminary analysis shows that the maximum and minimum wind attack angle tends to decrease gradually with an increase in the wind speed.

### Wind tunnel and field verification of the lodging CWS model

#### Wind tunnel simulation verification of the lodging CWS model

The results of the wind tunnel simulation of lodging resistance during 2013–2015 are shown in Table 3.

**Table 3:** Wind tunnel simulation experiments for lodging resistance during 2013–2014.

Variety	Canopy height (cm)	Lodging CTF (N)	lodging MCWS (m/s)	Ventilation coefficient	lodging SCWS-1 (m/s)	lodging SCWS-2 (m/s)	Wind speed at 10 m (m/s)
2013-2014(First planting)							
Yumai 18	80±1.14a	4.69±1.03ab	7.05±0.72b	0.26c	5.14c	6.90cd	15.75cd
Zhoumai 18	72±1.79bc	5.19±0.63ab	8.92±0.68ab	0.28b	5.77b	8.06b	18.74b
Aikang 58	69±1.50c	7.51±1.04a	10.27±0.23a	0.23d	7.14a	9.28a	21.75a
Pingan 6	77±1.91ab	4.74±0.85ab	8.94±0.50ab	0.33a	5.29c	7.39c	18.23b
Zhengmai9023	80±1.15a	4.44±1.04ab	7.41±0.87b	0.32a	4.99c	7.30c	16.66c
Caizhi 9998	75±1.08b	4.30±0.66b	6.82±0.84b	0.23d	5.12c	6.64d	15.34d
2013-2014(Second planting)							
Yumai 18	79±1.23a	3.79±0.66b	8.29±0.66ab	0.32b	4.65d	6.80c	15.55c
Zhoumai 18	72±0.94bc	6.69±1.48a	10.61±1.45a	0.27c	6.56b	9.02a	20.98a
Aikang 58	69±1.06c	7.65±0.66a	10.82±0.81a	0.26cd	7.21a	9.72a	22.76a
Pingan 6	78±0.87a	6.06±1.30a	10.44±1.30a	0.36a	5.93b	9.25a	21.20a
Zhengmai 9023	78±0.94a	4.30±1.67ab	7.23±1.12b	0.32b	5.00d	7.39bc	16.95bc
Caizhi 9998	74±0.41b	5.66±0.44ab	7.58±0.93b	0.24d	5.93b	7.79b	18.02b
2014–2015							
Wenmai 6	82±1.41a	6.63±1.51a	7.54±0.41d	0.23a	6.01c	7.81c	17.75e
Yumai 18	80±1.22ab	6.82±0.83a	8.02±0.34cd	0.24a	6.20bc	8.14bc	18.58cde
Zhoumai 26	79±1.91abc	7.52±1.27a	9.30±0.26b	0.24a	6.56bc	8.62bc	19.70bcd
Aikang 58	70±1.01e	8.91±1.49a	10.38±0.83a	0.23a	7.71a	10.02a	23.41a
Bainong 418	71±0.75de	7.02±1.23a	8.87±0.28b	0.23a	6.78b	8.81b	20.53b
Bainong 419	75±2.68cd	7.50±1.10a	8.93±0.18b	0.23a	6.77b	8.79b	20.30bc
Zhonglian 2	76±1.02bc	6.74±0.83a	8.66±0.45bc	0.24a	6.36bc	8.27bc	19.04bcde
Zhengmai 9023	82±1.10a	6.70±1.02a	7.64±0.68d	0.23a	6.05c	7.86c	17.85de
Average	76±1.26	6.144±1.04a	8.73±0.68	0.26	6.08	8.25	19.02

Note: The ‘lodging MCWS’ is the critical wind speed for wheat lodging in the wind tunnel. The ‘lodging SCWS-1’ is the lodging CWS calculated by the model, *k*_*p*_ = 0.75. The ‘lodging SCWS-2’ indicates the lodging CWS from the model and the ventilation coefficient. (4) Wind speed at 10 m is the lodging CWS at 10 m above ground based on the model.

Wind tunnel test results in different years showed that Aikang58 had the strongest resistance to stem lodging, and the lodging CWS was 22.64 m/s; followed by Zhoumai 26, Bainong 419 and Bainong 418, with the lodging CWS of 19.70–20.85 m/s; Yumai 18, Caizhi 9998 and Zhengmai 9023 had the weakest lodging resistance, and the lodging CWS was 16.62–16.81 m/s. This results were consistent with the field performance of these varieties. The lodging CTF was positively correlated with the actual wind speed measured in the wind tunnel (MCWS), the ground wind speed of lodging calculated by the model (SCWS-1), the ground wind speed of lodging determined by the model and the ventilation coefficient (SCWS-2) and the wind speed at 10 m above ground (with correlation coefficients of 0.641^**^, 0.972^**^, 0.866^**^, and 0.855^**^, respectively). Moreover, the ‘lodging MCWS’ also showed a positive correlation with the ‘lodging SCWS-1’ and ‘lodging SCWS-2’, and the wind speed at 10 m above ground (with correlation coefficients of 0.721^**^, 0.898^**^, and 0.903^**^, respectively). Due to the penetration and loss of the wind to the wheat population, the ‘lodging SCWS-1’ was significantly smaller than the ‘lodging MCWS’, but they were consistent following calibration with the ventilation coefficient.

#### Field experimental verification of the lodging CWS model

The lodging CTF was measured from 10 days after flowering during 2013–2014, and the determination time was on May 9, 16, 23, and 30, respectively. The test of lodging CTF for 2014–2015 started at wheat flowering on April 22, April 29, May 6, May 13, May 20, and May 27. To facilitate a comparative analysis, we selected four dates during the filling stage, the results of which are shown in Table 4.

**Table 4:** The experiment of lodging resistance in the field from 2013 to 2015.

Variety	Canopy height(cm)	Lodging CTF(Newton/m)	Lodging CWS(m/s)	Lodging CTF(Newton/s)	Lodging CWS(m/s)	Lodging CTF(Newton/m)	Lodging CWS(m/s)	Lodging CTF(Newton/m)	Lodging CWS(m/s)
		2014/05/09		2014/05/16		2014/05/23		2014/05/30	
Zhoumai18	79±1.89 a	10.85±0.69 b	17.03b	9.56±0.33 b	15.98c	8.93±0.19 d	15.45c	10.16±0.20b	16.48c
Akang58	70±2.46b	14.89±0.64 a	22.11a	13.73±1.04 a	21.41a	13.14±0.17 a	20.94a	12.77±0.49 a	20.64a
Zhoumai26	77±2.73ab	12.06±0.54 b	18.37b	10.58±0.67 b	17.20bc	11.34±0.01 b	17.80b	11.18±0.38 ab	17.68bc
Bainong418	71±2.28ab	12.67±0.97 ab	20.17ab	10.79±0.28 b	18.62b	10.73±0.31bc	18.57b	10.82±0.36 b	18.64b
Bainong419	75±3.87ab	11.19±0.64 b	18.04b	10.20±0.56 b	17.22bc	10.12±0.18 c	17.16bc	10.45±0.93 b	17.43bc
Zhonglian 2	76±1.31ab	11.37±0.91 b	18.06b	9.80±0.08 b	16.76bc	8.49±0.14 d	15.61c	10.03±0.63 b	16.96bc
Average	74±2.43	12.17±0.73	18.96	10.78±0.49	17.87	10.46±0.17	17.59	10.90±0.50	17.97
		2015/5/6		2015/5/13		2015/5/20		2015/5/27	
Zhoumai18	77±1.37 a	10.93±0.88 a	17.54bc	10.39±0.09 a	17.10bc	9.51±1.19 a	16.36b	11.14±1.23a	17.71b
Akang58	70±1.92 b	13.46±1.21 a	21.01a	11.48±1.00 a	19.40a	13.11±1.22 a	20.74a	12.79±1.12 a	20.48a
Zhoumai26	79±1.82 a	10.73±0.99 a	16.88c	11.35±1.19 a	17.36bc	11.25±0.95 a	17.29b	11.41±1.11 a	17.41b
Bainong418	75±2.42 ab	12.51±1.22 a	19.17ab	11.03±1.52 a	18.00ab	10.49±1.65 a	17.56b	10.69±1.59 a	17.72b
Bainong419	70±0.15 b	11.47±0.92a	19.29ab	10.49±1.21 a	18.45ab	11.74±1.24 a	19.52a	10.60±1.07 a	18.54b
Zhonglian 2	77±1.44 a	9.84±1.02 a	16.54c	8.89±0.83 a	15.72c	9.24±0.99 a	16.03b	11.21±0.72 a	17.65b
Average	75±1.52	11.49±1.04	18.41	10.60±0.97	17.67	10.89±1.21	17.92	11.31±1.14	18.25

Notice: The main parameters are: the wind angle of attackθ: -33°; the height of the ground equivalent lodging EWS = canopy height +1.0 m; the ventilation coefficient*α*: 0.1; the apparent roughness: 0.16 m; the lodging CWS adjustment coefficient (*k*):0.85; probe resistance coefficient (*k*_*p*_):0.75; gust factor *β*:1.59. The lodging CWS is the standard wind speed (instantaneous extreme wind speed), i.e. the wind speed at 10 m from the ground.

The results of field trials showed that Aikang58 had the strongest resistance to stem lodging from the flowering stage to full maturity in the two experimental years, and the average lodging CWS was 20.84 m/s; followed by Bainong 419 and Bainong 418, with the lodging CWS of 18.21 m/s and 18.56 m/s respectively. Zhoumai 18 and Zhoumai 26 with the lodging CWS of 16.71 m/s and 17.50 m/s respectively. The lodging CTF and CWS values were consistent between the years and among the different wheat varieties. Both were high at the early filling stage, and gradually decreased until maturity, followed by a slight increase thereafter. The field experiment results were basically consistent with the wind tunnel experiment results.

## Discussion

### Model of lodging CWS and optimizing its parameters

#### Basic characteristics of the wind field at the ground layer

The stability of the atmosphere is related to the observation time, the weather conditions and the wind speed. During the daytime, the atmosphere was mainly unstable, while it was predominately stable during the evening (Fig 4A–4C). During rainy days or persistent strong winds, *Ri* was relatively small, suggesting an unstable or neutral state of the atmosphere (Fig 4C). These results are consistent with those from a previous study of tropical rain forests in Xishuangbanna [38].

#### Wind speed profile

The wind speed profiles below 10 m were similar among the various times and wind speed conditions. The wind speed increased with height, where the change followed an exponential or logarithmic distribution with an *R*^2^ of 0.97. The distribution of wind speed profiles at the ground layer of the field was not affected by atmospheric conditions; thus, the influence of the atmosphere can be ignored when calculating wind speeds at heights below 10 m at the ground layer of the field [36].

#### Apparent roughness of wheat fields

The roughness of field crops is generally considered to be related to characteristics of the crop canopy and atmospheric stability. Because it is difficult to accurately determine the displacement height of the zero plane under the condition of the field, in this study, we used the apparent roughness to represent the height where the wind speed was zero at the ground layer, which is the “displacement of the zero plane + the roughness of the field crops”. Our results indicate that for strong winds, the apparent roughness decreases with an increase in the wind speed, and the relationship was exponential with an *R*^2^ varying from 0.916 to 0.926 (Fig 5). In the field, and at a crop canopy height of 0.7–0.8 m, the minimum standard wind speed that can disturb the crop canopy is about 2.0–2.5 m/s. The apparent roughness ranged from 0.10 to 0.24 m for wind speeds of 8.0–12.0 m/s. The apparent roughness values of wheat fields measured in this paper are significantly lower than those reported in the literature[36]. The main reason for this is related to the wind speed conditions when the roughness measurements were made. The apparent roughness in this paper is measured with strong wind but the values in the literature with weak wind. The results are consistent with the observation results of Liu [39] and Mao[40]. The apparent roughness of wheat field is not only related to the stability of the crop canopy structure and the atmospheric layer, but it is also related to the observed wind speed. This phenomenon is mainly related to the flexibility of the crop stems. The apparent roughness decreases with an increase in the wind speed, and eventually stabilizes at a minimum value. Thus, the magnitude of wind speed must be taken into account when measuring and calculating the apparent roughness of field crops. The apparent roughness was positively correlated with wind speed, where the lodging CWS increased by 2% with every 0.01 m decrease in the apparent roughness. The apparent roughness was generally 0.10–0.24 m during strong winds, and was 0.16 m in this study.

#### Wind attack angle

The attack angle is related to the observation time, the weather conditions and the height of the observation (see Fig 6A–6C and Table 3). Generally, the attack angle at night was negative, and it was positive during the day. Compared with sunny weather, the negative attack angle increased significantly on cloudy and rainy days, and the positive angle of attack decreased significantly (Fig 6A–6C). The phenomenon of wheat lodging occurring at night may be related to the attack angle. The wind attack angle exhibited an instantaneous variation and it decreased rapidly with an increase in the interval of observation. However, the range of the attack angle was much larger than that reported in the general literature, which may be related to the flexible structure of the crop canopy.

Due to the downward spread of wind at the ground layer of a field, wind can penetrate the canopy and cause lodging. Thus, the actual wind speed experienced by a wheat stem is larger than just the horizontal wind speed, that is, the wind speed impinging on the wheat stem = wind speed/cos *θ*. As lodging occurs instantaneously, and the wind angle of attack is related to the observation time and height, the attack angle is the maximum moving average of the attack angle over three seconds within 10-minute records of wind speed at 1.0 m above ground. To be conservative, the average of the top 25% of the “maximum moving average of the attack angle in three seconds” was used for the model. The lodging CWS decreased by 0.45% with every degree of increase in the negative attack angle.

#### Height of the ground-equivalent lodging CWS

The height of the ground-equivalent lodging wind speed is *H* = 1+(*h*_*c*_ -*z*_*0*_). The lodging CWS decreased by 2.2% with every 0.10 m decrease in the height. The value of this parameter is similar to that found in studies that used models of wheat lodging [1]. However, the canopy height of wheat in China is generally between 0.70 and 0.85 m, and some differences in the canopy heights exist among the different varieties.

#### Ventilation coefficient of wheat canopies

The ventilation coefficient of a wheat canopy depends on the number of main stems per unit area of the considered wheat population. The number of main stems (i.e. the panicle number) in the high-yield wheat population of China is generally 615–675 panicles/m^2^. According to our preliminary analysis, the ventilation coefficient at 2/3 of canopy height is 0.10–0.30 (for a canopy width of 1.0 m). The horizontal distribution of wind speed in the wheat canopy follows an exponential law, and the wind speed in the canopy decreases rapidly with the increase of the distance between the canopy and the edge of the population. The result is consistent with that of Wong et al [41]. The coefficient was smaller in the early growth stage of wheat than in the mature stage. The coefficient was set to 0.10–0.15 in our model because of the difficulty of accurate determination under the field conditions.

### Wind tunnel and field verification of the lodging CWS model

#### wheat population lodging CTF determination method

The accurate determination of the lodging CTF of wheat population is the basis for calculating the lodging CWS based on the Bernoulli equation. The lodging CTF measuring instrument used in this paper is a new instrument based on the electronic measuring device of crop lodging resistance[24]). The instrument still uses a dynamometer as the lodging CTF measuring component, and a ball guideway as the dynamometer horizontal moving component. However, the rotary positioning disc is used to replace ball joints to change the bearing of the dynamometer, and the combination of the circular scale rod and steel fork is used to replace the field fixation of the multi-functional tripod for the measuring device. Compared with the original device, the new instrument has the following four improvements. (1) The conversion of the horizontal and vertical positions of the dynamometer is faster and more accurate. (2) The measurement range of the lodging CTF is wider (0.10 m to 1.0 m above ground). (3) The canopy height and gravity center height can be measured directly by using the circular scale rod. (4) With three tooth steel fork as the fixed part of the measuring device, the field fixation of the instrument is faster and more convenient.

#### Wind tunnel verification of the lodging CWS model

Our results show that lodging CWS is significantly and positively correlated with the CTF; the correlation coefficient was 0.86 and 0.91 in 2014 and 2015, respectively, suggesting that it is feasible to calculate the lodging CWS by measuring the CTF. As wind can penetrate the wheat population, the actual wind pressure on the wheat stems is less than the wind pressure that can be generated by the field wind speed. Therefore, it is necessary to calibrate the Bernoulli equation with a ventilation coefficient when calculating the wind speed of lodging resistance for wheat.

#### Field experimental verification of the lodging CWS model

There was a significant correlation between the lodging instantaneous CWS measured by wind tunnel and field test in the same period (middle stage of wheat grouting) of 5 same wheat varieties (Zhoumai 18, Zhoumai 26, Aikong58, Bainong 418 and Bainong 419), with a correlation coefficient of 0.963^***^. The results of the field trials were consistent with those of the wind tunnel simulation.The results of the field lodging tests were significantly higher than those of the lab tests, which were mainly due to the difference in the environments for the wheat growth and test conditions. However, the lodging CWS obtained by the existing model was much larger than the value of general wheat varieties investigated. There were two main reasons for this phenomenon. The first was the friction resistance caused by existing population lodging thrust probe. To understand the influence of the probe’s friction resistance on the simulation results, we designed a new type of low-friction resistance probe. Compared with the new low-friction resistance probe, the measurement result of the existing population thrust probe was more than 25% higher. Second, the "radial width of wheat population" used in the determination of the lodging CTF exceeds the width of wheat population that can be penetrated by canopy wind speed. Due to the limitation of field environmental conditions, the population lodging thrust can only be measured by pushing forward pressure towards the population, so the lodging CTF must be corrected when calculating the lodging CWS. Where, the lodging CTF deviation caused by the friction resistance of the probe was corrected by the friction resistance coefficient *K*_*P*_ of the probe, *K*_*P*_ = 1- ((CTF_Existing probe_ -CTF_Low resistance probe_)/CTF_Low resistance probe_), *K*_*P*_ takes 0.75–1.0 depending on the resistance of the probe used. The lodging CTF deviation caused by the radial width of wheat population was corrected by the adjustment coefficient *k* of the population thrust, and *k* is generally 0.85. Aikang 58 exhibited the highest lodging resistance, followed by Bainong 418, Bainong 419, and Zhoumai 18 (Tables 3–4). The same was also seen in the field observations, indicating that the model is reliable for calculating the lodging CWS.

#### Field investigation and verification of the lodging CWS model

To explore the influence of wind speed and rainfall on wheat lodging, we conducted two surveys. For the first time, we used literature information to investigate the meteorological factors of 52 time’s large-scale wheat lodging in 2007–2015[42]. Large-scale wheat lodging in China can be classified into three types: strong winds, persistent rainfall, and strong winds with heavy rainfall, which account for 8%, 19% and 73% of all lodging events, respectively. Without rain, a wind speed above 16.75 m/s can lead to large-scale wheat lodging. The second time, on May 23, 2017, a large-scale wheat lodging occurred in the Huanghuai wheat area of China. We conducted field surveys on the lodging of wheat in 11 counties (cities) in Zhengzhou, Xinxiang and Jiaozuo, and conducted statistical analysis on meteorological factors. The results also showed that under the condition of no rainfall, a wind speed below 17.5–17.9 m /s would not cause large areas of wheat lodging. Two survey results are similar, revealing that the critical wind speed of general wheat lodging in China is about 17.0–18.0 m/s. Of course, the lodging wind speed is also related to the considered wheat variety and its growth stage.

**Fig 7:**
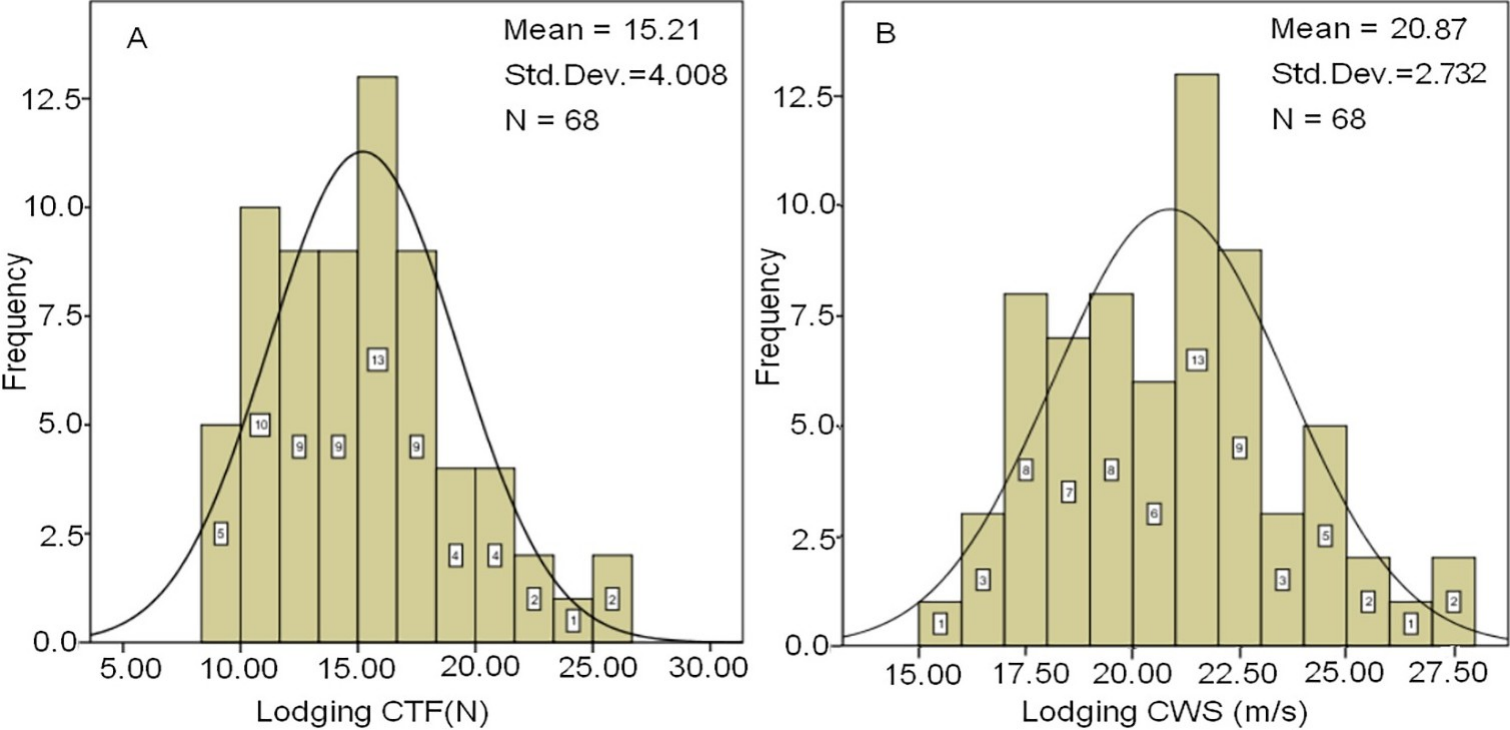
The lodging resistance distribution of common winter wheat varieties in China's Huanghuai wheat area. A. The lodging CTF distribution of winter wheat population; B. The lodging CWS (instantaneous extreme wind speed) of winter wheat population.

To understand the coincidence between the calculated lodging CWS of this model and the actual value of wheat varieties, the lodging CTF and lodging CWS of 68 winter wheat varieties in China's Huanghuai wheat area or regional test materials (hereinafter referred to as varieties) measured in the field from 2011 to 2016 were statistically analyzed (Fig 7).

The maximum of the lodging CTF of 68 varieties (*K*_*p*_ was 0.75, the same below) was 26.21 N, the minimum 8.3 N, and the average 15.21 N±4.008N (Fig 7A). Among them, there were 5 varieties with CTF below 10N, accounting for 7.4%; 27 varieties with 10N to 15N, accounting for 41.1%; 24 varieties with 15N to 20N, accounting for 38.3%; 9 varieties with more than 20N, accounting for 13.2%. At present, the canopy height of wheat varieties is about 0.75 m. Under the condition that the canopy height is 0.75m and other parameters are the same, the lodging CWS of the 68 varieties was calculated by using this model. The results showed that the minimum of the lodging CWS was 15.62 m/s, the maximum was 27.62m/s, and the average was 20.87 m/s±2.732 m/s (Fig 7B). Among them, there were 33 varieties with the lodging CWS below 21.0m/s, accounting for 48.5%; 21 varieties with 21.0 to 23.0m/s, accounting for 30.9%; and 13 varieties with 23.0m/s or above, accounting for 19%. The calculation results are basically consistent with the lodging CWS of the current wheat varieties.There is a significant correlation between the lodging CTF and the lodging instantaneous CWS in 68 wheat varieties, with a correlation coefficient of 0.997^***^. This result further indicates that the model and parameter setting are appropriate.

In this paper, wind speed and its related factors were studied, where the ventilation coefficient and the wind attack angle were introduced in our model, so that the model could more fully reflect the relationship between wheat lodging and meteorological factors. The lodging resistance of wheat depends on many factors such as plant height, height of gravity, plant stem strength, plant morphology, stem chemical composition, planting density, developmental period, etc [3,12–19]. These factors often have complex mutual constraints, such as plant height, plant stem strength, plant morphology, if these simple correlation factors are directly used to evaluate the lodging resistance of wheat, it is often difficult to accurately and truly evaluate the lodging resistance of wheat. On the contrary, if the black box theory is used, many factors, which are related to the lodging resistance of wheat but their real effects and their correlation with each other are difficult to be simply analyzed, can be included in a black box. The lodging evaluation process can be greatly simplified by studying from the perspective of the whole, namely "the lodging CTF of wheat population".

Compared with the stalk strength measurement method, this method for measuring the lodging resistance are similar, and the essence is the comparison of the bending strength of the stalk [9]. Compared with the lodging index method [8], there are four main characteristics: (1) The lodging CTF of wheat population in the field is used as an evaluation index, which can eliminate bias when calculating wheat lodging resistance by considering only the stalk strength, the stalk breaking strength, the plant height, planting density, etc. and the complex interactions that may exist between these evaluation indicators. (2) The measurement process has no obvious damage to the normal growth of wheat population evaluated, and is a non-destructive evaluation method for lodging resistance. (3) The method can not only determine the stem lodging resistance of wheat, but also its resistance to root lodging. (4) The model uses the lodging CWS as an indicator, and the result is related to the wind that causes the natural lodging of wheat lodging, which helps people to intuitively understand and compare the lodging resistance of different varieties.

Due to a limitation of available measured wind attack angles and ventilation coefficients, accurately determining their values will undoubtedly improve the accuracy of future results. Furthermore, many investigations have showed that lodging arising from strong winds coupled with rainfall is the main type, and how to calculate the lodging wind speed under the condition of rainfall is an important problem that needs to be studied urgently. Although the mechanism and cause of wheat stem lodging are different from root lodging, the analysis and research methods of wheat stem lodging are also applicable to root lodging.

## Conclusions

In this paper, the characteristics of the wind field related to wheat lodging were investigated, a new device was proposed for directly measuring the critical thrust force of wheat population lodging resistance in the field, and a wind speed model of the stem lodging resistance of wheat was studied using a wind tunnel simulation and field experiments. The lodging CTF was significantly and positively correlated with the ‘lodging MCWS’, the ‘lodging SCWS-1’, the ‘lodging SCWS-2’, and the wind speed at 10 m. The lodging CWS can be calculated based on the lodging CTF and such parameters as the apparent roughness, wind attack angle, ventilation coefficient, and the height of the equivalent wind speed. The model can eliminate the biases associated with evaluating lodging resistance as it considers only a few indicators, and thus can be widely employed to assess lodging resistance, the selection of extension regions for varieties of wheat, and the evaluation of lodging factors in the field.

## Supporting information

S1 File Figs 4–6 Data1. (ZIP)

S2 File Figs 4–6 Data2. (ZIP)

S3 File Figs 4–6 Data3. (ZIP)

S4 File Fig 5 Data1. (XLS)

S5 File Fig 5 Data2. (XLS)

S6 File Fig 5 Data3. (XLS)

S7 File Table 2 Data. (XLS)

S8 File Fig 7 Data. (XLS)
